# Specific Paucity of Unmyelinated C-Fibers in Cutaneous Peripheral Nerves of the African Naked-Mole Rat: Comparative Analysis Using Six Species of Bathyergidae

**DOI:** 10.1002/cne.23133

**Published:** 2012-04-24

**Authors:** Ewan S Smith, Bettina Purfürst, Tamara Grigoryan, Thomas J Park, Nigel C Bennett, Gary R Lewin

**Affiliations:** 1 Department of Neuroscience, Max-Delbrück Center for Molecular MedicineD 13125 Berlin, Germany; 2 Electron Microscopy Core Facility, Max-Delbrück Center for Molecular MedicineD 13125 Berlin, Germany; 3 Department of Cancer Research, Max-Delbrück Center for Molecular MedicineD 13125 Berlin, Germany; 4 Laboratory of Integrative Neuroscience, Department of Biological Sciences, University of Illinois at ChicagoChicago, Illinois 60612; 5 Department of Zoology and Entomology, Mammal Research Institute, University of Pretoria5100 Pretoria, South Africa

**Keywords:** naked mole-rat, C-fiber, A-fiber, Remak bundle, nociceptor, Bathyergidae

## Abstract

In mammalian peripheral nerves, unmyelinated C-fibers usually outnumber myelinated A-fibers. By using transmission electron microscopy, we recently showed that the saphenous nerve of the naked mole-rat (*Heterocephalus glaber*) has a C-fiber deficit manifested as a substantially lower C:A-fiber ratio compared with other mammals. Here we determined the uniqueness of this C-fiber deficit by performing a quantitative anatomical analysis of several peripheral nerves in five further members of the Bathyergidae mole-rat family: silvery (*Heliophobius argenteocinereus*), giant (*Fukomys mechowii*), Damaraland (*Fukomys damarensis*), Mashona (*Fukomys darlingi*), and Natal (*Cryptomys hottentotus natalensis*) mole-rats. In the largely cutaneous saphenous and sural nerves, the naked mole-rat had the lowest C:A-fiber ratio (∼1.5:1 compared with ∼3:1), whereas, in nerves innervating both skin and muscle (common peroneal and tibial) or just muscle (lateral/medial gastrocnemius), this pattern was mostly absent. We asked whether lack of hair follicles alone accounts for the C-fiber paucity by using as a model a mouse that loses virtually all its hair as a consequence of conditional deletion of the β-catenin gene in the skin. These β-catenin loss-of function mice (β-*cat LOF* mice) displayed only a mild decrease in C:A-fiber ratio compared with wild-type mice (4.42 compared with 3.81). We suggest that the selective cutaneous C-fiber deficit in the cutaneous nerves of naked mole-rats is unlikely to be due primarily to lack of skin hair follicles. Possible mechanisms contributing to this unique peripheral nerve anatomy are discussed. J. Comp. Neurol. 520:2785–2803, 2012. © 2012 Wiley Periodicals, Inc.

The axons present in the peripheral nerves of mammals can originate from motor neurons, pre- and postganglionic autonomic neurons, and sensory neurons with their cell bodies in the trigeminal and dorsal root ganglia. The majority of axons in peripheral nerves are sensory axons, and these can be split into two main groups: myelinated A-fibers and unmyelinated C-fibers. Large-diameter afferent Aβ-fibers are involved in mechano- and proprioception, whereas smaller-diameter Aδ-fibers are either mechanoreceptors (D-hair receptors) or nociceptors (Aδ-mechanonociceptors), some of which are also thermosensitive (Smith and Lewin,^2009^). C-fibers are divided into different groups according to their sensitivity to different noxious, chemical, and thermal stimuli, but there are also silent C-fibers, which are activated only following sensitization, and a group of low-threshold C-fibers (Olausson et al.,^2010^), which have not been classified as nociceptors (for a detailed review of different sensory fiber types see Lynn,^1994^; Lewin and Moshourab,^2004^; Smith and Lewin,^2009^; Dubin and Patapoutian,^2010^). Unlike A-fibers, whose myelin sheath isolates individual fibers, unmyelinated C-fibers are grouped together in bundles by nonmyelinating Schwann cells, forming so-called Remak bundles.

The need to detect potentially damaging stimuli has been the selection pressure behind the evolution of nociceptors, and their importance for protective reflexes may explain the fact that C-fibers often outnumber A-fibers (Smith and Lewin,^2009^). This predominance of C-fibers over A-fibers is well documented for the saphenous nerve, which normally contains only cutaneous afferents innervating the medial knee, lower leg, and foot (Zimmermann et al.,^2009^). The saphenous nerve is frequently used in an *in vitro* skin nerve preparation to characterize sensory afferent fiber properties (Reeh,^1986^; Koltzenburg and Lewin,^1997^; Koltzenburg et al.,^1997^; Milenkovic et al.,^2007^, ^2008^; Wetzel et al.,^2007^; Lechner and Lewin,^2009^). Measurements of the C:A-fiber ratio in the saphenous nerve by transmission electron microscopy have shown that C-fibers outnumber A-fibers with a ratio of ∼4:1 in a number of mammalian species: rat (*Rattus norvegicus*; Scadding,^1980^; Alpsan and Lal,^1980^; Lynn,^1984^; Jancso et al.,^1985^; Carter and Lisney,^1987^), mouse (*Mus musculus*; Stucky et al.,^2002^; Wetzel et al.,^2007^; Milenkovic et al.,^2007^; Stürzebecher et al.,^2010^), and dog (*Canis lupus familiaris*; Illanes et al.,^1990^). The sural nerve, which also contains predominantly cutaneous axons and innervates the lateral calf and foot (Peyronnard and Charron,^1982^; Lewin and McMahon,1991a, b), has also been observed to contain a C:A-fiber ratio of ∼4:1 in both humans and rabbits, *Oryctolagus cuniculus* (Ochoa and Mair,^1969^; Schwab et al.,^1984^).

In contrast to this generally conserved high C:A-fiber ratio, we recently found that the saphenous nerve in African naked-mole rats (*Heterocephalus glaber*) has a pronounced deficit in C-fibers, so that the C:A-fiber ratio is only ∼1.1:1 (Park et al.,^2008^). In the rat, C:A-fiber ratios lower than those measured from cutaneous nerves have been found in mixed nerves, which innervate both muscle and skin, such as the common peroneal and tibial nerves (Swett et al.,^1991^; Schmalbruch,^1986^), which have a C:A-fiber ratio of ∼2:1 (Schmalbruch,^1986^; Jenq et al.,^1987^). Even lower C:A-fiber ratios are observed in pure muscle nerves, e.g., the lateral and medial gastrocnemius nerves (Swett et al.,^1991^), in which C:A-fiber ratios of just ∼1.5:1 were measured (Jenq and Coggeshall,1984a, b, 1985a, b; Jenq et al.,^1984^).

Naked mole-rats are one of approximately 20 species in the African mole-rat family, the Bathyergidae, which have been split into six genera by using morphological and molecular techniques (Faulkes et al.,^2004^; Ingram et al.,^2004^; Kock et al.,^2006^; Deuve et al.,^2008^). All Bathyergidae are subterranean and feed on geophytes, the underground storage organs of plants. However, bathyergids occur in a wide range of soil types (from sand to fine clay) and range in body mass from ∼35 g (naked mole-rat) to ∼2 kg (Cape dune mole-rat, *Bathyergus suillus*), and their social organization varies from solitary (e.g., the silvery mole-rat, *Heliophobius argenteocinereus*) to eusocial (naked mole-rat; Bennett and Faulkes,^2000^); furthermore, naked mole-rats are unique in being naked and poikilothermic (Buffenstein and Yahav,^1991^) and have much longer life spans (∼30 years) than their body size would predict (Buffenstein,^2005^, ^2008^; Edrey et al.,^2011^).

A lack of fur in the naked mole-rat correlates with a complete lack of hair follicles other than infrequent large body hairs and facial vibrissae (Crish et al.,^2003^), which in other rodents, including the common mole-rat *Cryptomys hottentotus*, are innervated by both A- and C-fibers (Rice et al.,^1997^; Park et al.,^2003^). Naked mole-rats also have no sweat glands (Tucker,^1981^), which are also innervated by C-fibers (Fundin et al.,^1997^). Therefore, the naked mole-rat C-fiber deficit could be secondary to a loss of hair follicles and sweat glands.

We have performed a quantitative examination of peripheral nerve fibers that innervate a variety of tissues, to determine the degree to which the paucity of peripheral nerve C-fibers in the naked mole-rat is specific to cutaneous nerves and additionally performed a comparative study of six members of the African mole-rat family Bathyergidae. By examining a mouse model completely lacking hair follicles, we could also test the idea that lack of hair alone accounts for the paucity of C-fibers in naked mole-rats.

## MATERIALS AND METHODS

### Animals

Two animals each from six species of Bathyergidae were used for this study: naked mole-rat (*Heterocephalus glaber*, two males, ∼35 g, from T. Park's colony at the University of Illinois at Chicago); silvery mole-rat (*Heliophobius argenteocinereus*, one female, 200 g, and one male, 260 g, captured at Morogoro, Tanzania, permit from Tanzanian Nature Conservation Dar es Salaam); giant mole-rat (*Fukomys mechowii*, one female, 160 g, and one male, 215 g, captured in Chingola, Zambia, permit from the Department of Veterinary and Nature Conservation, Chingola, Zambia); Damaraland mole-rat (*Fukomys damarensis*, two males, 120 and 155 g, captured in Dordabis, Namibia, permit from the Department of Nature Conservation and Tourism, Namibia); Mashona mole-rat (*Fukomys darlingi*, one female, 65 g, and one male, 75 g, captured in Goromonzi, Zimbabwe, permit from the Department of National Parks and Wildlife Services, Harare, Zimbabwe); and Natal mole-rat (*Cryptomys hottentotus natalensis*, two females, 85 and 105 g, captured in Glengary, Natal, South Africa, permit from the Department of Nature Conservation, Ezemvelo, Kwa-Zulu, Natal, South Africa). These six species represent four of the six genera of Bathyergidae. All mole-rats, other than naked mole-rats, were captured in the wild, so determining the exact age was not possible, but all animals used were considered to be adults and were housed in cages as befitting their social nature: silvery and giant mole-rats singly and Damaraland, Mashona, and Natal mole-rats in small groups with food (sweet potato, carrot, and apple) available ad libitum. Naked mole-rats (aged approximately 5 years, e.g., young adults) were housed at the Max-Delbrück Center in Berlin, Germany, in cages connected by tunnels, which were contained within a humidified incubator (40% humidity, 28–30°C), and heated cables ran under at least one cage per colony to allow for behavioral thermoregulation. Food (sweet potato, banana, apple, and carrot) was available ad libitum.

Tissue-specific β-catenin (β-cat) loss-of-function (LOF) mutant mice were generated by crossing mice with loxP sites flanking exons 3–5 of the β-cat gene with mice expressing Cre recombinase under the *keratin 14* (K14) gene promoter as previously described (Huelsken et al.,^2001^). The strain generated were on a mixed 129 × C57Bl6 background and lack β-cat in the skin, tongue, and esophagus (*K14-Cre;β-cat LOF* mice). Hair follicle stem cells no longer differentiate into follicular keratinocytes, which produces a complete lack of hair follicles after approximately P30 (Huelsken et al.,^2001^). The mice used in this study were aged 43, 56, and 71 days, with wild-type littermates used as controls. The electron microscopic analysis was conducted by an experimenter blind to the genotype. Experiments were conducted under protocols approved by the German federal authorities (State of Berlin), and ethical clearance was also obtained to collect and perfuse the mole-rats by the Animal Use and Care Committee of the University of Pretoria (AUCC-060719-020 and AUCC 000418-006).

### Perfusion, dissection, and fixation

All animals were anesthetized with halothane (Sigma, St. Louis, MO) inhalation, except for naked mole-rats and mice (Ketavet [Pfizer] coadministered with the muscle relaxant Rompun [Bayer] intraperitoneally) and then intracardially perfused with 0.1 M phosphate-buffered saline (PBS; pH 7.4), followed by freshly prepared 4% paraformaldehyde in 0.1 M PBS. Saphenous, sural, common peroneal, tibial, lateral gastrocnemius, and medial gastrocnemius nerves were dissected from both legs and postfixed in 4% paraformaldehyde/2.5% glutaraldehyde in 0.1 M PBS for 3 days (only saphenous and tibial nerves were taken from mice). No major differences were observed in the anatomy of the sciatic nerve branches in comparison with what has been published for the rat (Schmalbruch,^1986^; Swett et al.,^1991^). For the saphenous nerve, branching is sometimes observed at the knee joint (Zimmermann et al.,^2009^), so saphenous samples were always taken from above the knee. In *H. argenteocinereus*, saphenous branching was observed in all cases but was always higher than the knee joint; samples were always taken before this branching event.

### Electron microscopy

After treatment with 1% OsO_4_ for 2 hours, each nerve was dehydrated in a graded ethanol series and propylene oxide and then embedded in Poly/Bed 812 (Polysciences, Warrington, PA). Semithin sections were stained with toluidine blue. Ultrathin sections (70 nm) were contrasted with uranyl acetate and lead citrate. Sections were examined with a Zeiss 910 electron microscope, and digital images were taken with a high-speed slow-scan CCD camera (Proscan) at an original magnification of ×1,600. Three ultrathin sections were taken from at least two nerves, usually three (nerve loss or damage sometimes occurred during either dissection or the embedding procedure), and on each ultrathin section four images (18.2 × 18.2 μm) were taken. Myelinated and unmyelinated axons were counted in these areas in iTEM software (Olympus Soft Imaging Solutions, Münster, Germany) and normalized to the whole nerve. The original images were of resolution sufficient to use the digital zoom function of the iTEM program to allow the counting and measuring of small C-fiber axons. For calculating C:A-fiber ratios (C-fiber count/A-fiber count), an average was taken for each ultrathin section per nerve, and the averages were used for calculating significant differences between species using the unpaired *t*-test. Both axonal and fiber diameter were measured for A-fibers, which allowed for the calculation of g-ratios (Rushton,^1951^). C-fiber diameter was also measured along with the number of C-fibers per Remak bundle. Histograms of A- and C-fiber diameter were plotted in Prizm 5.0b (GraphPad Software, Williston, VT). Differences between C:A-fiber ratios across bathyergids were assessed by using a one-way ANOVA and Bonferroni's post hoc test, and unpaired *t*-tests were conducted for comparisons between wild-type and transgenic mice. All measurements are displayed as mean ± SEM.

### Photomicrographs

Electron microscopic photomicrographs for figures were made in Adobe Illustrator software without changing brightness or contrast.

### Body surface area calculation

Body surface area (BSA) was calculated based on the formula: BSA = K × W^2/3^, originally proposed by Meeh (1879), where W = body weight in grams, and K is a shape constant for a given species. The 2/3 power of the weight and K values have been recalculated for several species, and thus the following formulae were used for calculating BSA: mouse, 20 × W^0.42^ (Dawson,^1967^); rat, 12.54 × W^0.6^ (Lee,^1929^); cat, 9.6 × W^0.67^ (Vaughan and Adams,^1967^); and dog, 10 × W^0.67^ (Price and Frazier,^1998^). To our knowledge, such calculations have not been made for any of the species of mole-rat used in this study, so the formula used for the mouse has been applied. However, it should be noted that the mouse has a longer tail and large pinnae in comparison with most mole-rat species, so the formula provides an approximation of BSA in these species.

## RESULTS

### C:A-fiber ratios are low in naked mole-rat cutaneous nerves

**Figure 1:**
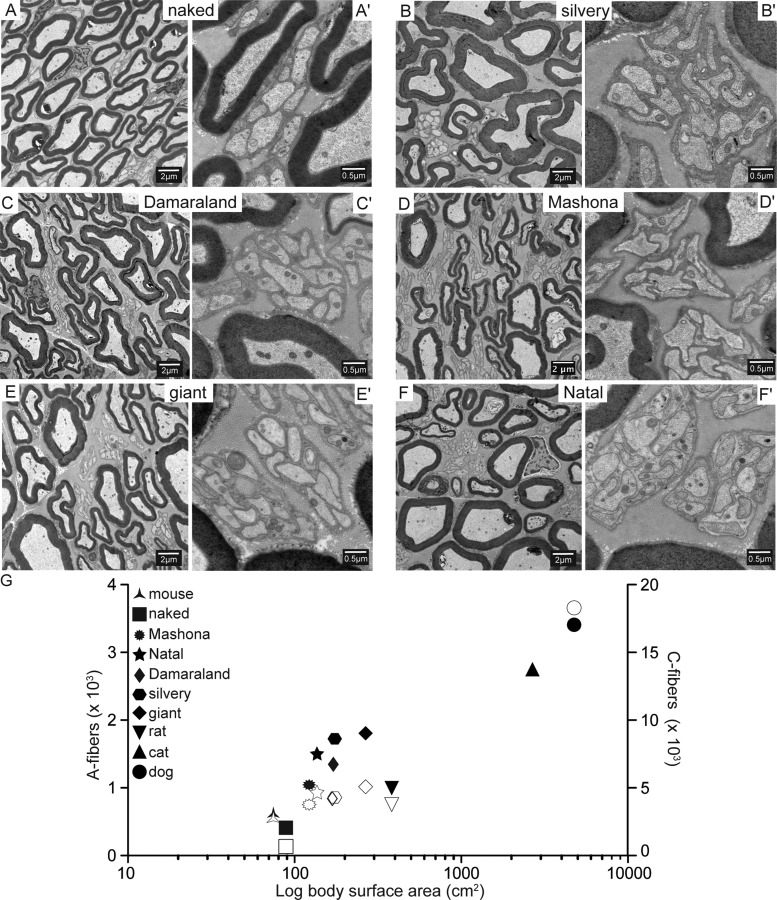
Naked mole-rats have a significantly lower C:A-fiber ratio in saphenous nerves compared with other Bathyergidae. Example electron micrographs and quantification for each left panel image showing A-fibers (A), C-fibers (C), and Remak bundles (R) for: **A**, naked (A = 31, C = 51 and R = 8); **B**, silvery (A = 12, C = 36, and R = 7); **C**, Damaraland (A = 30, C = 81, and R = 19); **D**, Mashona (A = 25, C = 170, and R = 27); **E**, giant (A = 21, C = 79, and R = 12); and **F**, Natal (A = 19, C = 93, and R = 15) mole-rats. **A′–F**′ are high-magnification images demonstrating C-fiber structure. **G:** Comparison of A- and C-fiber count with body surface area (BSA) in several different species. Solid symbols correspond to A-fibers and open symbols to C-fibers. For species not examined in this study, data were taken from: mouse (Milenkovic et al.,^2007^; Wetzel et al.,^2007^; Park et al.,^2008^), rat (Scadding,^1980^; Lynn,^1984^; Carter and Lisney,^1987^), cat (Sherrington,^1894^; Gasser and Grundfest,^1939^; Douglass et al.,^1934^), and dog (Illanes et al.,^1990^). Scale bars = 2 μm in A–F; 0.5 μm in A′–F′.

**Figure 2:**
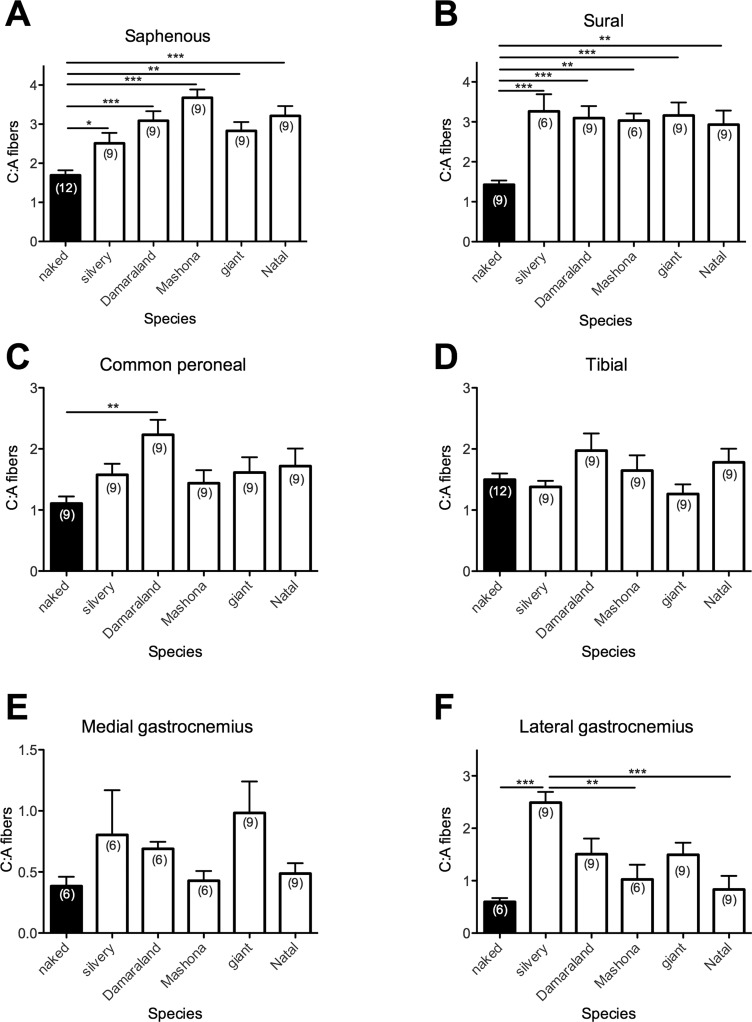
The low C:A-fiber ratio in naked mole-rats is largely restricted to cutaneous nerves. **A,B:** Naked mole-rats have a significantly lower C:A-fiber ratio in cutaneous saphenous and sural nerves compared with all other species. Values for C:A-fiber ratio in common peroneal and tibial nerves (**C,D**), which innervate both skin and muscle, as well as medial gastrocnemius and lateral gastrocnemius nerves (**E,F**), which innervate only muscle, are largely similar across all species. Numbers in parentheses refer to the number of ultrathin sections from which average C:A-fiber ratios were calculated. *P < 0.05, **P < 0.01, ***P < 0.001.

We first set out to determine whether the very low C:A-fiber ratio that we had previously observed in the largely cutaneous saphenous nerve of the naked mole-rat (Park et al.,^2008^) is consistent in nerves that innervate other targets in this species and how A- and C-fiber counts compare across bathyergids. A- and C-fibers were first counted (Tables 1, 2), and an analysis of C:A-fiber ratios was performed for saphenous nerves from naked mole-rats and five other Bathyergidae species, which showed ratios ranging from 2.5:1 (silvery) to 3.7:1 (Mashona), all of which were significantly greater than the 1.7:1 ratio observed for the naked mole-rat (Figs. 1, 2A; *P* < 0.05 for the silvery mole-rat, *P* < 0.01 for the giant mole-rat, and *P* < 0.001 for all other species; example electron micrographs are shown in Fig. 1A–F). A similar pattern was observed in the other cutaneous nerve examined, the sural nerve. The mean C:A-fiber ratio in the naked mole-rat sural nerve was significantly lower, 1.4:1, compared with 2.9–3.3:1 in the other species (Fig. 2B; *P* < 0.01 for Mashona and Natal mole-rats, *P* < 0.001, for all other species).

**Table 1:** Summary of Saphenous, Sural, and Common Peroneal Nerve Fiber Data for Each Species^1^

Species	Common name	Sex and weight (g)	Nerve	Nerve area (μm^2^)	A-fibers	A-fiber diameter (μm)	g-Ratio	C-fibers	C-fiber diameter (μm)
*Heterocephalus glaber*	Naked mole-rat	M (∼30)	SA	5,696 ± 1,016 (4)	413 ± 54 (4)	3.17 ± 0.04 (472)	0.63 ± 0.003 (472)	682 ± 101 (4)	0.48 ± 0.006 (509)
		M (∼30)							
*Heliophobius argenteocinereus*	Silvery mole-rat	M (260)	SA	51,814 ± 4,978 (3)	1,711 ± 48 (3)	4.55 ± 0.09 (204)	0.53 ± 0.005 (204)	4,271 ± 362 (3)	0.59 ± 0.008 (534)
		F (200)							
*Fukomys damarensis*	Damaraland mole-rat	M (120)	SA	18,998 ± 866 (3)	1,348 ± 88 (3)	3.06 ± 0.08 (209)	0.53 ± 0.005 (209)	4,200 ± 361 (3)	0.41 ± 0.005 (521)
		M (155)							
*Fukomys darlingi*	Mashona mole-rat	M (75)	SA	14,844 ± 1,284 (3)	1,018 ± 33 (3)	3.12 ± 0.07 (247)	0.57 ± 0.006 (247)	3,759 ± 344 (3)	0.47 ± 0.006 (591)
		F (65)							
*Fukomys mechowii*	Giant mole-rat	M (215)	SA	43,029 ± 6,531 (3)	1,804 ± 32 (3)	3.86 ± 0.09 (290)	0.59 ± 0.005 (290)	5,081 ± 289 (3)	0.50 ± 0.009 (547)
		F (160)							
*Cryptomys hottentotus natalensis*	Natal mole-rat	F (85)	SA	25,445 ± 2,355 (3)	1,495 ± 208 (3)	3.82 ± 0.08 (261)	0.59 ± 0.006 (261)	4,674 ± 320 (3)	0.48 ± 0.005 (613)
		F (105)							
*Heterocephalus glaber*	Naked mole-rat	M (∼30)	SU	6,983 ± 356 (3)	430 ± 31 (3)	3.43 ± 0.06 (234)	0.61 ± 0.004 (234)	618 ± 147 (3)	0.57 ± 0.007 (505)
		M (∼30)							
*Heliophobius argenteocinereus*	Silvery mole-rat	M (260)	SU	29,512 ± 3,579 (2)	1,101 ± 90 (2)	4.62 ± 0.08 (167)	0.55 ± 0.005 (167)	3,520 ± 191 (2)	0.55 ± 0.009 (510)
		F (200)							
*Fukomys damarensis*	Damaraland mole-rat	M (120)	SU	9,617 ± 1,095 (3)	505 ± 46 (3)	3.42 ± 0.08 (203)	0.59 ± 0.005 (203)	1,517 ± 80 (3)	0.51 ± 0.005 (522)
		M (155)							
*Fukomys darlingi*	Mashona mole-rat	M (75)	SU	6,291 ± 828 (2)	370 ± 36 (2)	3.03 ± 0.08 (226)	0.58 ± 0.006 (226)	1,127 ± 183 (2)	0.58 ± 0.006 (632)
		F (65)							
*Fukomys mechowii*	Giant mole-rat	M (215)	SU	12,459 ± 1,988 (3)	577 ± 34 (3)	3.73 ± 0.09 (269)	0.6 ± 0.004 (269)	1,805 ± 257 (3)	0.60 ± 0.004 (554)
		F (160)							
*Cryptomys hottentotus natalensis*	Natal mole-rat	F (85)	SU	11,702 ± 1,252 (3)	506 ± 8 (3)	3.82 ± 0.08 (285)	0.64 ± 0.005 (285)	1,475 ± 231 (3)	0.46 ± 0.005 (582)
		F (105)							
*Heterocephalus glaber*	Naked mole-rat	M (∼30)	CP	21,365 ± 1,397 (3)	1310 ± 74 (3)	3.48 ± 0.07 (203)	0.61 ± 0.003 (203)	1,469 ± 291 (3)	0.50 ± 0.006 (439)
		M (∼30)							
*Heliophobius argenteocinereus*	Silvery mole-rat	M (260)	CP	56,627 ± 1,589 (3)	1,814 ± 57 (3)	4.75 ± 0.1 (194)	0.6 ± 0.006 (194)	2,916 ± 319 (3)	0.65 ± 0.01 (420)
		F (200)							
*Fukomys damarensis*	Damaraland mole-rat	M (120)	CP	56,562 ± 5,656 (3)	2,653 ± 166 (3)	3.17 ± 0.09 (213)	0.63 ± 0.007 (213)	5,990 ± 1,114 (3)	0.54 ± 0.006 (450)
		M (155)							
*Fukomys darlingi*	Mashona mole-rat	M (75)	CP	56,313 ± 6,018 (3)	2,241 ± 188 (3)	3.84 ± 0.11 (210)	0.62 ± 0.007 (210)	3,224 ± 341 (3)	0.50 ± 0.007 (571)
		F (65)							
*Fukomys mechowii*	Giant mole-rat	M (215)	CP	67,420± 5,497 (3)	2,429 ± 224 (3)	4.27 ± 0.11 (197)	0.62 ± 0.005 (197)	3,207 ± 1058 (3)	0.50 ± 0.008 (376)
		F (160)							
*Cryptomys hottentotus natalensis*	Natal mole-rat	F (85)	CP	60,677 ± 5,624 (3)	2,159 ± 134 (3)	4.26 ± 0.12 (171)	0.65 ± 0.01 (171)	3,730 ± 295 (3)	0.54 ± 0.007 (508)
		F (105)							

1 Raw data summarizing the name and weight of species used in this study; the size and number (in parentheses) of the nerves used; and the number of A- and C-fibers counted, A- and C-fiber diameters, and g-ratios. SA, saphenous nerve; SU, sural nerve; CP, common peroneal.

**Table 2:** Summary of Tibial, Medial Gastrocnemius, and Lateral Gastrocnemius Nerve Fiber Data for Each Species^1^

Species	Common name	Sex and weight (g)	Nerve	Nerve area (μm^2^)	A-fibers	A-fiber diameter (μm)	g-Ratio	C-fibers	C-fiber diameter (μm)
*Heterocephalus glaber*	Naked mole-rat	M (∼30)	TI	33,704 ± 691 (4)	2,024 ± 96 (4)	3.39 ± 0.07 (216)	0.61 ± 0.004 (216)	3,090 ± 178 (4)	0.60 ± 0.007 (506)
		M (∼30)							
*Heliophobius argenteocinereus*	Silvery mole-rat	M (260)	TI	215,371 ± 28,163 (3)	6,480 ± 557 (3)	4.90 ± 0.11 (158)	0.6 ± 0.006 (158)	8,791 ± 728 (3)	0.67 ± 0.011 (354)
		F (200)							
*Fukomys damarensis*	Damaraland mole-rat	M (120)	TI	128,119 ± 12,949 (3)	5,065 ± 119 (3)	3.52 ± 0.11 (197)	0.63 ± 0.008 (197)	10,027 ± 1,463 (3)	0.57 ± 0.008 (461)
		M (155)							
*Fukomys darlingi*	Mashona mole-rat	M (75)	TI	130,826 ± 1,140 (3)	5,564 ± 106 (3)	3.80 ± 0.1 (191)	0.66 ± 0.006 (191)	9,729 ± 1,307 (3)	0.56 ± 0.01 (494)
		F (65)							
*Fukomys mechowii*	Giant mole-rat	M (215)	TI	127,176 ± 3,251 (3)	4,520 ± 373 (3)	4.42 ± 0.11 (164)	0.62 ± 0.006 (164)	5,782 ± 1,420 (3)	0.56 ± 0.01 (346)
		F (160)							
*Cryptomys hottentotus natalensis*	Natal mole-rat	F (85)	TI	95,324 ± 14,025 (3)	3,835 ± 125 (3)	4.07 ± 0.09 (199)	0.66 ± 0.006 (199)	6,736 ± 975 (3)	0.55 ± 0.008 (536)
		F (105)							
*Heterocephalus glaber*	Naked mole-rat	M (∼30)	MG	2,529 ± 309 (2)	101 ± 14.6 (2)	4.03 ± 0.09 (170)	0.63 ± 0.005 (170)	36.9 ± 8.9 (2)	0.65 ± 0.02 (86)
		M (∼30)							
*Heliophobius argenteocinereus*	Silvery mole-rat	M (260)	MG	17,228 ± 1,428 (2)	282 ± 27.7 (2)	6.15 ± 0.28 (49)	0.67 ± 0.009 (49)	226.9 ± 63.9 (2)	0.73 ± 0.04 (78)
		F (200)							
*Fukomys damarensis*	Damaraland mole-rat	M (120)	MG	9,672 ± 1,838 (2)	251 ± 97 (2)	5.09 ± 0.21 (71)	0.59 ± 0.009 (71)	162 ± 38 (2)	0.57 ± 0.02 (78)
		M (155)							
*Fukomys darlingi*	Mashona mole-rat	M (75)	MG	10,502 ± 879.7 (2)	250.4 ± 30.2 (2)	5.11 ± 0.18 (92)	0.65 ± 0.006 (92)	110.7 ± 6.65 (2)	0.51 ± 0.02 (56)
		F (65)							
*Fukomys mechowii*	Giant mole-rat	M (215)	MG	13,194 ± 1,641 (3)	219.4 ± 13.9 (3)	5.78 ± 0.19 (67)	0.61 ± 0.01 (67)	219.7 ± 35.63 (3)	0.75 ± 0.02 (170)
		F (160)							
*Cryptomys hottentotus natalensis*	Natal mole-rat	F (85)	MG	10,091 ± 1,209 (3)	187 ± 20.3 (3)	6.45 ± 0.23 (75)	0.66 ± 0.006 (75)	95.1 ± 40.3 (3)	0.50 ± 0.015 (72)
		F (105)							
*Heterocephalus glaber*	Naked mole-rat	M (∼30)	LG	2,690 ± 144 (2)	124 ± 9 (2)	3.78 ± 0.1 (137)	0.61 ± 0.006 (137)	77 ± 6 (2)	0.43 ± 0.01 (48)
		M (∼30)							
*Heliophobius argenteocinereus*	Silvery mole-rat	M (260)	LG	10,940 ± 1,941 (3)	331 ± 35 (3)	4.32 ± 0.15 (102)	0.6 ± 0.008 (102)	830 ± 137 (3)	0.69 ± 0.01 (532)
		F (200)							
*Fukomys damarensis*	Damaraland mole-rat	M (120)	LG	9,376 ± 1,187 (3)	297 ± 97 (3)	4.03 ± 0.12 (157)	0.65 ± 0.01 (157)	533 ± 309 (3)	0.49 ± 0.007 (332)
		M (155)							
*Fukomys darlingi*	Mashona mole-rat	M (75)	LG	11,106 ± 531 (2)	411 ± 15 (2)	3.92 ± 0.14 (129)	0.64 ± 0.006 (129)	428 ± 153 (2)	0.55 ± 0.02 (198)
		F (65)							
*Fukomys mechowii*	Giant mole-rat	M (215)	LG	12,196 ± 1,620 (3)	557 ± 202 (3)	3.58 ± 0.12 (162)	0.59 ± 0.005 (162)	989 ± 463 (3)	0.43 ± 0.006 (365)
		F (160)							
*Cryptomys hottentotus natalensis*	Natal mole-rat	F (85)	LG	9,169 ± 1,525 (3)	253 ± 68 (3)	4.88 ± 0.2 (90)	0.67 ± 0.007 (90)	251 ± 140 (3)	0.49 ± 0.02 (161)
		F (105)							

1 Raw data summarizing the name and weight of species used in this study; the size and number (in parentheses) of the nerves used; and the number of A- and C-fibers counted, A- and C-fiber diameters, and g-ratios. TI, tibial; MG, medial gastrocnemius; LG, lateral gastrocnemius.

A low C:A-fiber ratio could be the result of either a paucity of C-fibers or an overabundance of A-fibers. With data from this and previous studies, we plotted BSA against saphenous nerve A-/C-fiber counts for different species and observed a positive correlation between A-/C-fiber number and BSA. However, the naked mole-rat did not have a particularly high number of A-fibers compared with its BSA, whereas the C-fiber count was exceptionally low with respect to BSA (Fig. 1G), suggesting that a C-fiber deficit as opposed to more A-fibers underlies the low C:A-fiber ratio observed. A similar result was observed when plotting A- and C-fiber counts against BSA for the sural nerve (data not shown).

For the rat, previous studies have shown that the mixed common peroneal and tibial nerves have lower C:A-fiber ratios than the cutaneous saphenous and sural nerves, ∼2:1 compared with ∼4:1 (Scadding,^1980^; Alpsan and Lal,^1980^; Lynn,^1984^; Schwab et al.,^1984^; Jenq and Coggeshall,1984a, b, 1985a, b; Schmalbruch,^1986^; Peyronnard et al.,^1986^; Carter and Lisney,^1987^; Jenq et al.,^1987^). Here we also found that the C:A-fiber ratios were lower in the common peroneal and tibial nerves (Fig. 2C,D) compared with the saphenous and sural nerves in all species studied (Fig. 2A,B), except for the naked mole-rat. Indeed, in the naked mole-rat, the C:A-fiber ratio was found to be uniformly about 1.5:1 in the saphenous, sural, common peroneal, and tibial nerves (Fig. 2A–D). Although the naked mole-rat C:A-fiber ratio was significantly lower than that of all other species in the saphenous and sural nerves (Fig. 2A,B), in the common peroneal nerve it was only significantly lower than that of the Damaraland mole-rat (1.1:1 compared with 2.2:1, *P* < 0.001; Fig. 2C), and the C:A-fiber ratio in tibial nerves was similar across all species, ∼1.5:1 (Fig. 2D). Similarly, the naked mole-rat C:A-fiber ratio in both the medial and the lateral gastrocnemius nerves, although low (0.4:1 and 0.6:1, respectively), was not unique in being significantly lower compared with the ratio in some other bathyergids. For the medial gastrocnemius nerve, the C:A-fiber ratios are <1:1 in all species, and, for the lateral gastrocnemius nerve, the naked mole-rat C:A-fiber ratio of 0.6:1 is significantly lower than that of the silvery mole-rat (2.5:1, *P* < 0.001), but the Natal (0.8:1) and Mashona mole-rats (1:1) also have significantly smaller C:A-fiber ratios than the silvery mole-rat (*P* < 0.001 and *P* < 0.01, respectively; Fig. 2F).

In considering the data by species, rather than by nerve, there is a clear trend in all species other than the naked mole-rat for saphenous and sural nerves to have much higher C:A-fiber ratios than in mixed and muscle nerves (∼3:1 compared with ∼1.5:1 in common peroneal and tibial, ∼1:1 lateral gastrocnemius, and ∼0.5:1 for medial gastrocnemius). However, for the naked mole-rat, there is little difference among saphenous, sural, common peroneal, and tibial, all of which have a C:A-fiber ratio of ∼1.5:1. Examining the data from mixed and muscle nerves in all species shows two clear trends: the common peroneal and tibial nerves have higher C:A-fiber ratios than either of the pure muscle nerves, lateral and medial gastrocnemius nerves. However, we did note that the C:A-fiber ratios in the lateral gastrocnemius nerve were approximately twice as large as those in the medial gastrocnemius nerve in all the species examined.

### A-fibers in bathyergid peripheral nerves

**Figure 3:**
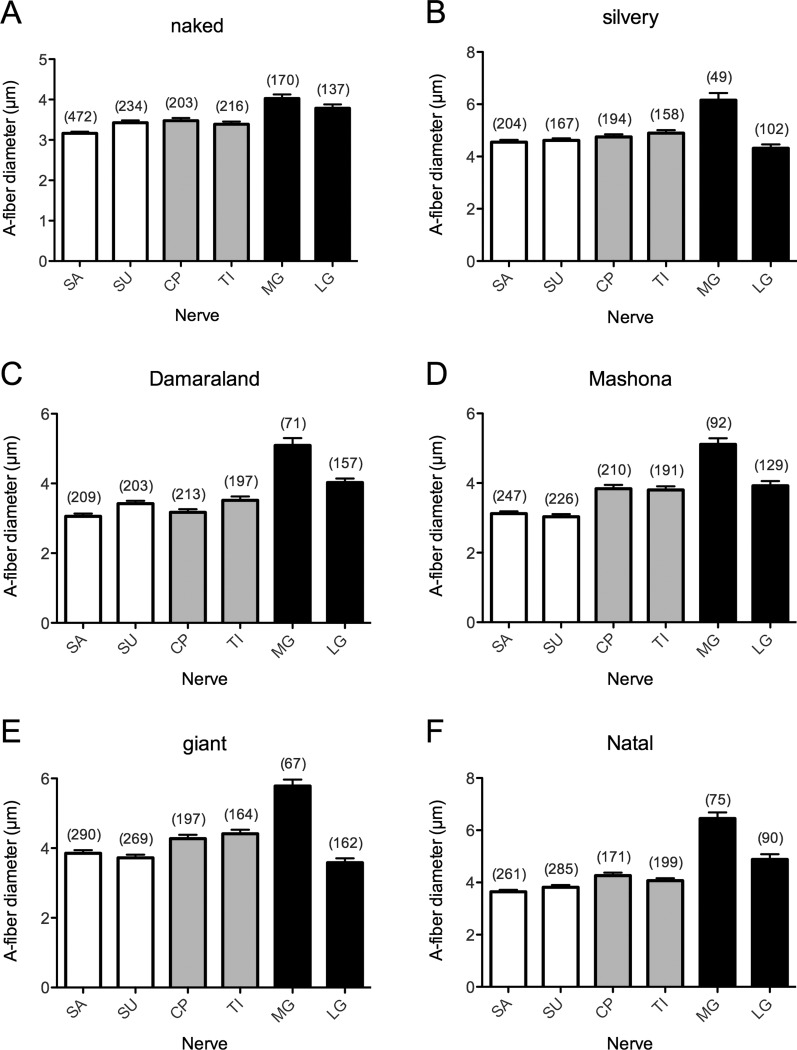
A-fibers in muscle-innervating nerves have the largest diameter. In Mashona (**D**), giant (**E**), and Natal (**F**) mole-rats, nerves innervating skin and muscle (gray bars) have A-fibers of a larger average diameter than those that are only cutaneous (white bars), a trend not apparent in naked (**A**), silvery (**B**), or Damaraland (**C**) mole-rats. In naked (A), Damaraland (C), Mashona (D), and Natal (F) mole-rats, the muscle-innervating medial gastrocnemius and lateral gastrocnemius nerves (black bars) contained A-fibers with an average diameter larger than the diameters of the other four nerves examined. For silvery (B) and giant (E) mole-rats, this is true only for the medial gastrocnemius nerve. In all species, A-fibers of the medial gastrocnemius nerve had the largest average diameter. SA, saphenous nerve; SU, sural nerve; CP, common peroneal; TI, tibial; MG, medial gastrocnemius; LG, lateral gastrocnemius. Numbers in parentheses refer to the number of A-fibers measured.

Across the six bathyergid species, there was a general trend for larger species to possess larger-diameter A-fibers on average than smaller species for each nerve. The silvery mole-rat, being the largest species examined, most often possessed the largest mean average diameter of A-fibers, but this was not true for either the medial or the lateral gastrocnemius nerves (Fig. 3, Tables 1, 2). Because of the presence of large-diameter type Ia afferent fibers and motor neurons, it has long been known that nerves innervating muscle have, on average, larger diameters than cutaneous nerves (Sherrington,^1894^; Boyd and Davey,^1968^). This fact was confirmed in this study for members of the Bathyergidae family, A-fiber diameter being larger in those nerves innervating muscle compared with skin, with the largest diameter being observed in the medial gastrocnemius nerve in all species (Fig. 3A–F, Tables 1, 2). Although less clear in the naked mole-rat, the myelin thickness, as measured by calculating the g-ratio (A-fiber axonal diameter/fiber diameter, Rushton,^1951^), was often slightly larger in the common peroneal, tibial, and medial and lateral gastrocnemius nerves compared with the cutaneous saphenous and sural nerves and most likely reflects an increased frequency of type Ia fibers in muscle nerves (Tables 1, 2).

**Figure 4:**
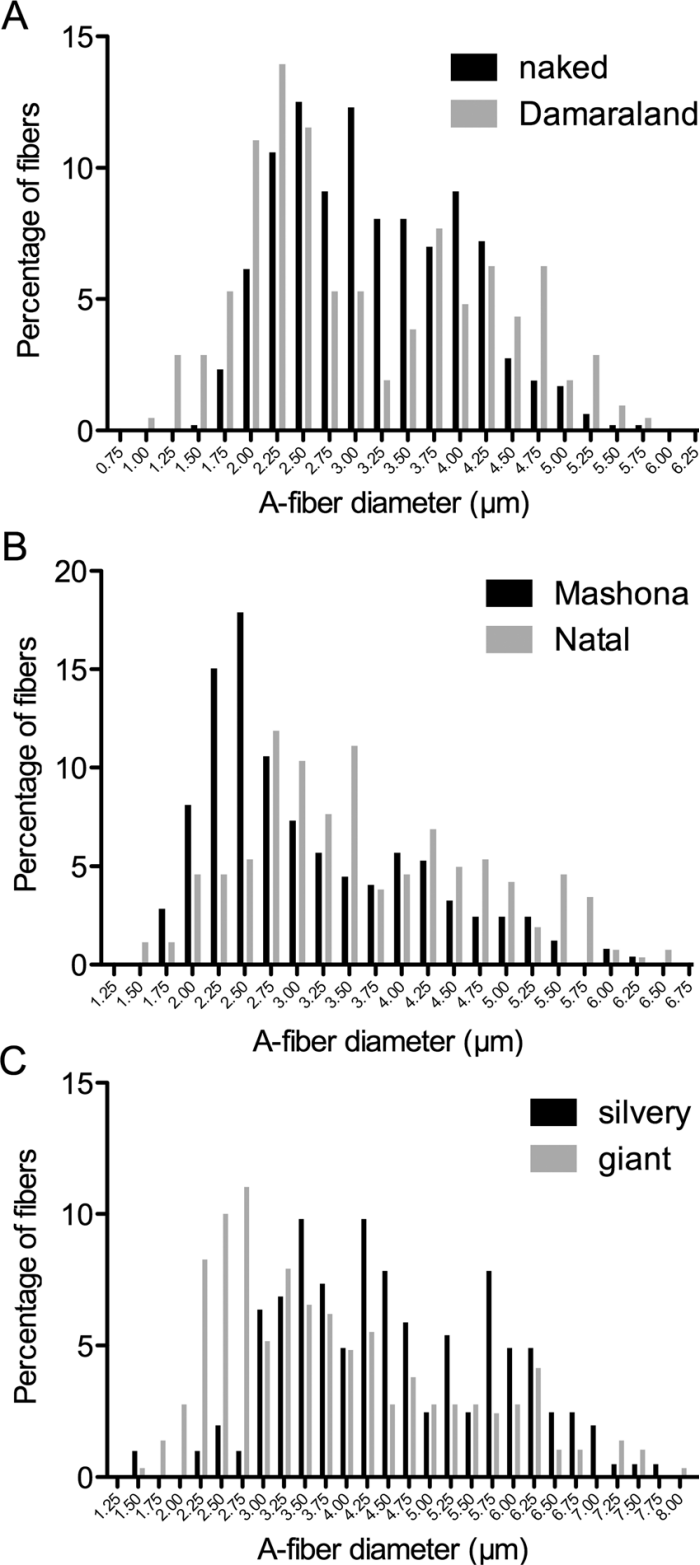
Histograms of A-fiber diameters in saphenous nerves in six species of bathyergid. **A:** Histogram for naked and Damaraland mole-rats. **B:** Histogram for Mashona and Natal mole-rats. **C:** Histogram for silvery and giant mole-rats. A-fiber diameters were binned into 0.25-μm bins, resulting in bimodal populations.

In the saphenous nerve, a histogram of A-fiber diameters shows a bimodal distribution for each species (Fig. 4A). A bimodal distribution is consistent with the presence of large-diameter Aα/β-fibers and smaller-diameter Aδ-fibers. Similar patterns were observed for A-fiber diameters in other nerves (data not shown). Mean A-fiber diameters for each nerve, from each species, are given in Tables 1 and 2.

### C-fiber anatomy in bathyergid peripheral nerves

**Figure 5:**
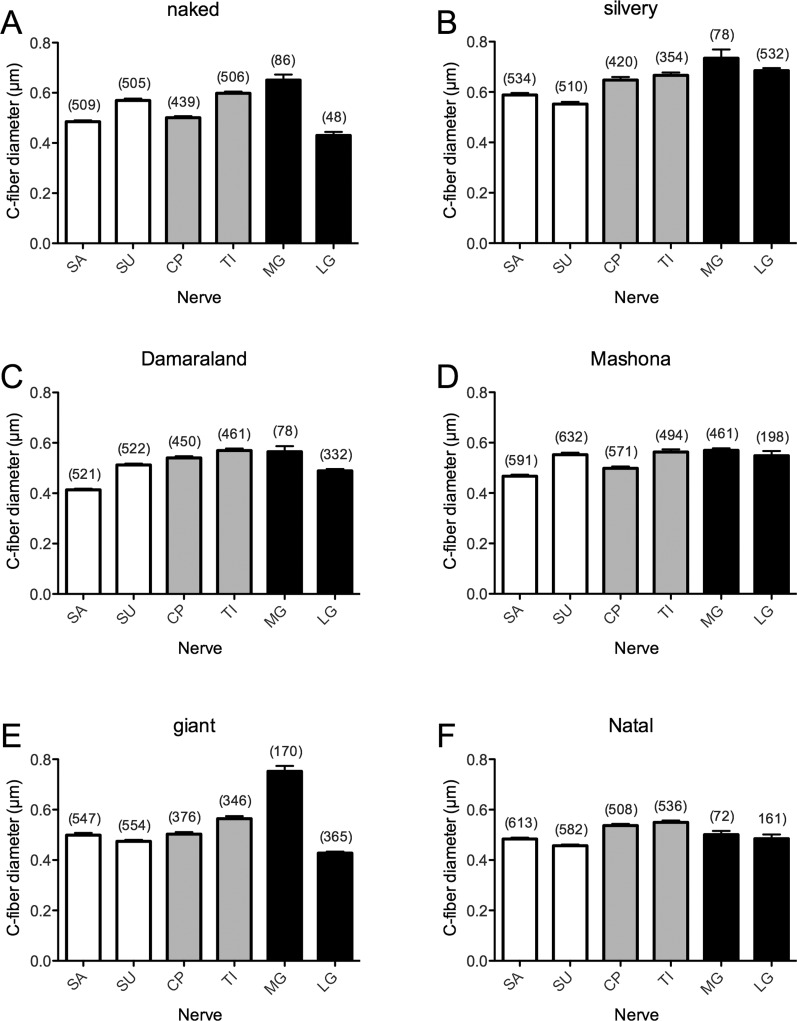
C-fiber diameters are generally smaller in cutaneous nerves. **A–F:** Average C-fiber diameter in six different nerves of the six Bathyergidae species examined. In many species, C-fibers in common peroneal and tibial (gray bars) and medial gastrocnemius and lateral gastrocnemius nerves (black bars) had diameters larger than those in the cutaneous saphenous and sural nerves (white bars), for example, in the silvery mole-rat (B). Tibial C-fibers always had a larger average diameter than in common peroneal nerves, and C-fiber diameters in medial gastrocnemius nerves were on average always larger than in lateral gastrocnemius nerves, often (naked, silvery, Mashona, and giant) having the largest average C-fiber diameter of all nerves examined. SA, saphenous nerve; SU, sural nerve; CP, common peroneal; TI, tibial; MG, medial gastrocnemius; LG, lateral gastrocnemius. Numbers in parentheses refer to the number of C-fibers measured.

**Figure 6:**
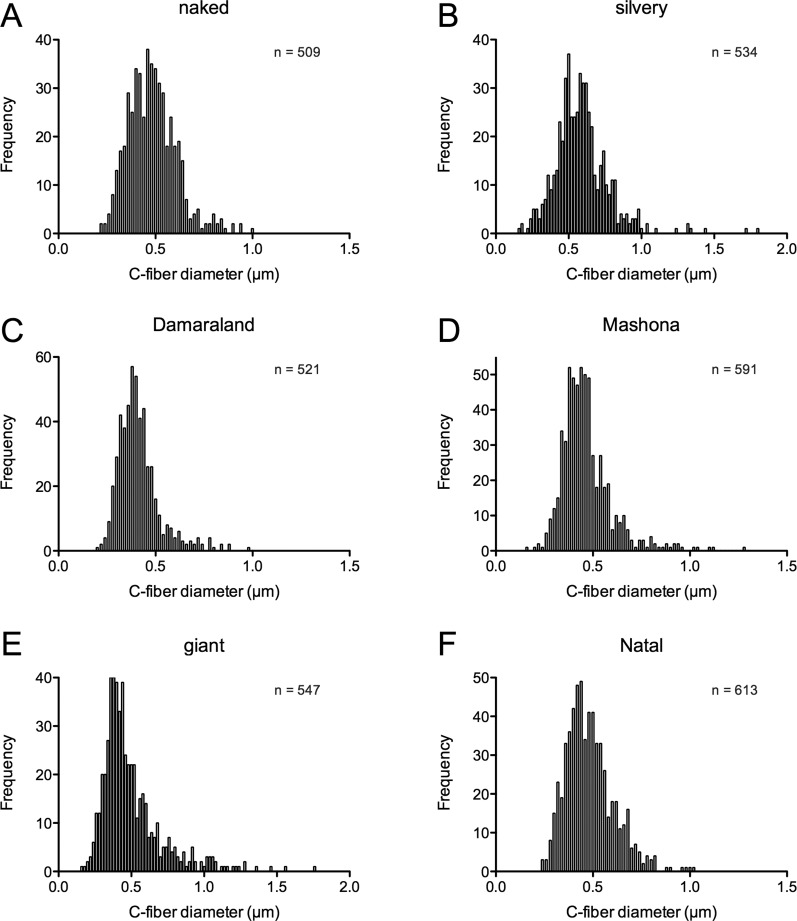
Histograms of C-fiber diameters in saphenous nerves in six species of bathyergid. **A–F:** C-fiber diameters were binned into 0.02-μm bins, which produced approximately unimodal distributions in the saphenous nerves of all species, although in most species there were a few outliers at the wider end of the diameter distribution, best observed in the two larger species examined, the silvery (B) and giant (E) mole-rats.

Across the six bathyergid species, there was a general trend for larger species to possess larger-diameter C-fibers than smaller species for each nerve. However, although the largest species examined in this study, the silvery mole-rat, often had the largest average C-fiber diameter, this was not true for every nerve examined (Tables 1, 2). Similar to the trends observed for A-fibers was a general trend for those nerves innervating both skin and muscle/just muscle (common peroneal, tibial, medial gastrocnemius, and lateral gastrocnemius) to have larger C-fiber diameters than those in nerves that are predominantly cutaneous (saphenous and sural; Fig. 5A–F, Tables 1, 2). Furthermore, as was observed with A-fibers, C-fibers in the medial gastrocnemius nerve tended to be of the largest diameter and were always larger than those of the lateral gastrocnemius. In contrast to A-fibers, C-fibers in all six nerves of all species were observed to be unimodal. An example is shown for the saphenous nerve (Fig. 6); data for other nerves are not shown.

**Figure 7:**
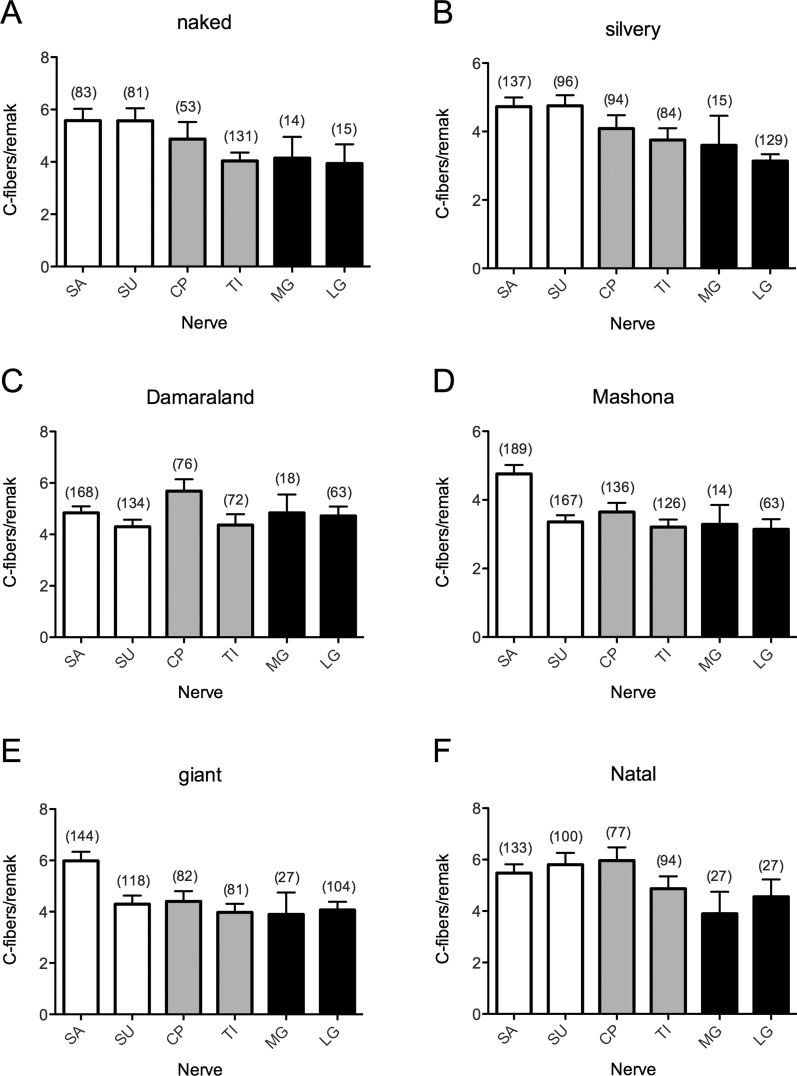
The number of C-fibers per Remak bundle is relatively conserved across nerves of bathyergids. **A–F:** The cutaneous saphenous and sural nerves (white bars) as well as the skin- and muscle-innervating common peroneal nerve (gray bar) averaged four to six C-fibers/Remak across all species, exceptions being the silvery common peroneal nerve (B) and Mashona sural and common peroneal nerves (D). The tibial nerve (gray bar) and muscle-innervating medial gastrocnemius and lateral gastrocnemius nerves (black bars) had a maximum of ∼4.5 C-fibers/Remak, the greatest variation across species being observed in the medial gastrocnemius and lateral gastrocnemius nerves. SA, saphenous nerve; SU, sural nerve; CP, common peroneal; TI, tibial; MG, medial gastrocnemius; LG, lateral gastrocnemius. Numbers in parentheses refer to the number of Remak bundles assessed.

Having observed a large deficit in C-fiber number in the saphenous and sural nerves of the naked mole-rat (Figs. 1G, 2A), we examined whether this could be explained by a lower number of C-fibers per Remak bundle. However, we observed that, in all species, there were on average four to six C-fibers/Remak in saphenous, sural, and common peroneal nerves, whereas in tibial, medial gastrocnemius, and lateral gastrocnemius nerves ∼4.5 C-fibers/Remak was the maximum density reached (Fig. 7).

### C:A-fiber ratios in hair follicle-deficient mice

**Figure 8:**
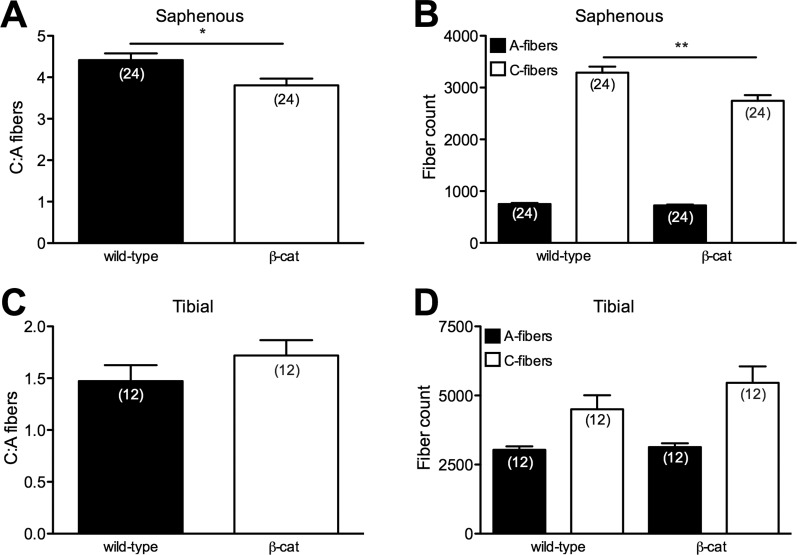
Mice lacking hair follicles have a small, cutaneous C-fiber loss. **A:** β-*Cat LOF* mice have a lower C:A-fiber ratio in saphenous nerves than wild-type littermates, which is due to a decreased total C-fiber number (**B**). There is no difference between genotypes in the C:A-fiber ratio in the tibial nerve (**C**), nor is there any difference between total fiber counts (D). Numbers in parentheses refer to the number of ultrathin sections, from which average C:A-fiber ratios were calculated. *P < 0.05, **P < 0.01.

Hair follicles receive both a myelinated and an unmyelinated fiber innervation (Rice et al.,^1997^; Li et al.,^2011^; Wende et al.,^2012^), and, although the few body hairs that naked mole-rats have are innervated in a manner similar to that of guard hairs in rats (Park et al.,^2003^), the cutaneous paucity of C-fibers observed could be connected to the relative lack of hair follicles in this species. To investigate this, we used a mouse model in which a conditional LOF mutation of β-cat in the skin was produced using a K14-promoter driven cre (β-*cat LOF* mice), which results in a complete lack of hair follicles and essentially naked mice after approximately P30. The loss of hair in this model is due to hair follicle stem cells no longer differentiating into follicular keratinocytes (Huelsken et al.,^2001^). Saphenous nerves from wild-type mice had a C:A-fiber ratio of 4.42, which was significantly greater than in β-*cat LOF* mice from the same litters (3.81, *P* < 0.05; Fig 8A). No significant difference was observed in the total number of A-fibers between genotypes, but, in β-*cat LOF* mice, a significant reduction in the total number of C-fibers was observed compared with controls (*P* < 0.01; Fig, 8B). In contrast, we observed no difference between genotypes in the largely noncutaneous tibial nerve in terms of C:A-fiber ratio or total fiber counts (Fig. 8C,D).

## DISCUSSION

By making a detailed and quantitative comparison of six, hind-leg-innervating, peripheral nerves in six species (four genera) from the African mole-rat family Bathyergidae, we conclude that naked mole-rats are unique among these bathyergids (and other mammals examined to date) in having a low C:A-fiber ratio in the cutaneous saphenous and sural nerves, whereas no such C-fiber deficit was observed in tibial, common peroneal, medial gastrocnemius, or lateral gastrocnemius nerves, which provide innervation to skeletal muscle.

### Low C:A-fiber ratio in naked mole-rat saphenous and sural nerves

Unmyelinated cutaneous C-fibers perform different functions in mammals, ranging from being polymodal nociceptors to thermoreceptors and even low-threshold mechanoreceptors (Olausson et al.,^2010^; Li et al.,^2011^; Wende et al.,^2012^). The ability to detect noxious stimuli is fundamental for an organism's survival and has presumably been the selection pressure behind the evolution of an elaborate repertoire of nociceptors in mammals to carry out this task (Kavaliers,^1988^; Walters,^1996^; Smith and Lewin,^2009^). C-fiber nociceptors usually outnumber A-fibers (predominantly non-nociceptors) in nerves innervating the skin, including the saphenous nerve (Scadding,^1980^; Alpsan and Lal,^1980^; Lynn,^1984^; Jancso et al.,^1985^; Carter and Lisney,^1987^; Illanes et al.,^1990^; Milenkovic et al.,^2007^; Wetzel et al.,^2007^; Park et al.,^2008^) and sural nerve (Ochoa and Mair,^1969^; Schwab et al.,^1984^; Jenq and Coggeshall,1984a, b, 1985a, b; Peyronnard et al.,^1986^). We have previously shown that, in comparison with other rodents, the naked mole-rat saphenous nerve has a C-fiber deficit, resulting in a very low C:A-fiber ratio (Park et al.,^2008^), which is confirmed in this study (Fig. 2A). By conducting a comparative study with other members of the bathyergid family, we can now conclude that this phenomenon is species specific; all other Bathyergidae species that we examined had significantly higher C:A-fiber ratios in the saphenous nerve (2.5–3.7:1). Comparing calculated body surface area to A-/C-fiber counts supports the hypothesis that the low C:A-fiber ratio in naked mole-rat saphenous nerves is due to a loss of C-fibers rather than an increase in A-fiber numbers (Fig. 1G). In addition, naked mole-rats are slightly larger than mice, and, although one would therefore expect higher total fiber counts in the naked mole-rat, this is not the case compared with the mice used in this study (A-fibers 412 ± 29 vs. 751 ± 20, and C-fibers 682 ± 55 vs. 3,289 ± 115 in the saphenous nerve), which again supports the hypothesis that the low C:A-fiber ratio observed in naked mole-rat cutaneous nerves is due to a C-fiber deficit as opposed to more A-fibers.

By examining branches of the sciatic nerve, which innervate different tissues (sural, largely skin; common peroneal and tibial, skin and muscle; medial gastrocnemius and lateral gastrocnemius, muscle; Schmalbruch,^1986^; Swett et al.,^1991^; Lewin and McMahon,1991a, b), we observed that the C-fiber deficit in naked mole-rats appears to be restricted to cutaneous nerves: naked mole-rats had a significantly lower C:A-fiber ratio in the sural nerve (1.4:1, compared with 2.9–3.3:1 for other species; Fig. 2B), whereas C:A-fiber ratios in the common peroneal, tibial, medial gastrocnemius, and lateral gastrocnemius nerves were more similar across all species (Fig. 2C–F) and more similar to those ratios previously observed in the rat (Jenq et al.,^1984^, ^1987^; Jenq and Coggeshall,1984a, b, 1985a, b; Schmalbruch,^1986^). An exception was the significantly lower C:A-fiber ratio in the common peroneal nerve compared with Damaraland mole-rats (Fig. 2C). The common peroneal nerve innervates both skin and muscle (Schmalbruch,^1986^), and it is possible that, in species with particularly high common peroneal C:A-fiber ratios, a larger proportion of the common peroneal axons innervates skin than in other species.

In humans, C-fiber innervation of the skin is very dense; receptive fields overlap, and this leads to spatial summation of noxious stimuli, which may well aid high-resolution stimulus localization (Jørum et al.,^1989^; Ochoa and Torebjörk,^1989^; Koltzenburg et al.,^1993^; Schmidt et al.,^1997^). Therefore, it might be expected, based on the C-fiber deficit observed in naked mole-rats, that naked mole-rats have a hypofunctional nociceptive system. Systematic examination of the nociceptive system in the naked mole-rat demonstrated that the animals have normal nocifensive responses to heat and mechanical stimuli but that they fail to respond behaviorally to certain chemical stimuli: acid, capsaicin, and histamine (Park et al.,^2008^; Smith et al.,^2010^; Brand et al.,^2010^). Isolated sensory neurons are, however, responsive to both capsaicin and histamine, and it is thought that unusual connectivity in the spinal cord and an endogenous lack of neuropeptides in C-fibers may explain the lack of nocifensive and scratching behavior evoked by these substances (Park et al.,^2008^; Smith et al.,^2010^). Interestingly, acid fails to activate naked mole-rat C-fiber nociceptors (Park et al.,^2008^), and, although one explanation for the C-fiber deficit would be that naked mole-rats lack acid-sensitive nociceptors, it has now been shown that acid insensitivity most likely is due to a variant of the voltage-gated sodium channel (Na_V_) Na_V_1.7, which is hypersensitive to acid block (Smith et al.,^2011^).

It should also be noted that some unmyelinated fibers are autonomic sympathetic fibers. In the rat, the contribution of sympathetic fibers has been measured to be sural 35%, common peroneal 27%, and tibial 40% of unmyelinated fibers (Schmalbruch,^1986^). Therefore, naked mole-rats might have lost sympathetic fibers as opposed to C-fiber afferent nociceptors. However, a mixture of the afferent and efferent C-fiber loss more likely is due to the dramatic C-fiber loss in cutaneous nerves, which could not be accounted for by loss of sympathetic efferent fibers alone. Interestingly, in congenital insensitivity to pain with anhidrosis (hereditary sensory and autonomic neuropathy 4, HSAN4), mutations in tyrosine receptor kinase A (TrkA), the receptor for nerve growth factor (NGF), result in subjects presenting with a total loss of C-fibers and lack of nociception, and they do not sweat due to hypotrophic, uninnervated sweat glands (Indo,^2009^). Moreover, a newly identified loss of function NGF mutation also results in a lack of nociception and anhidrosis (Carvalho et al.,^2011^), whereas a second NGF mutation, which causes a lack of nociception without anhidrosis (Einarsdottir et al.,^2004^), is proposed to be hypofunctional. Mice, in which either NGF or TrkA has been ablated, also show a severe C-fiber loss and lack normal nocifensive responses (Smeyne et al.,^1994^; Crowley et al.,^1994^). The fact that naked mole-rats have a hypofunctional nociceptive system (Park et al.,^2008^) as well as lacking sweat glands (Tucker,^1981^) raises the possibility that hypofunctional NGF-TrkA signaling might underlie the C-fiber deficit. However, if such hypofunctional NFG-TrkA signaling exists, then the effect must be restricted to cutaneous sensory afferents.

Another possible explanation for the cutaneous C-fiber deficit might simply be the lack of hair follicles, which are normally innervated by both A- and C-fibers (Fundin et al.,^1997^; Park et al.,^2003^). Therefore, the profound absence of hair follicles may result in an absence of C-fibers because they lack their normal cutaneous target. There are very few nonaquatic mammals lacking body hair; the hairless bat *Cheiromeles torquatus* being one example, but these animals do have hair on their undersides (Stephen Rossiter, Queen Mary University of London; personal communication). We therefore made use of a transgenic mouse model that completely lacks hair to model the situation in the naked mole-rat. In β-*cat LOF* mice, hair follicle stem cells fail to differentiate into follicular keratinocytes, producing a progressive hair loss resulting from a lack of hair follicles (Huelsken et al.^2001^). Interestingly, in the saphenous nerve, but not in the tibial nerve, we observed a decrease in the C:A-fiber ratio and total C-fiber number in adult β-*cat LOF* mice compared with wild-type littermates. These results suggest that a lack of hair follicles results in a mild cutaneous C-fiber deficit, but the fact that the saphenous C:A-fiber ratio in β-*cat LOF* mice is still more than double that of the naked mole-rat (3.81 vs. 1.69) suggests that mechanisms other than hair follicle loss are needed to explain the cutaneous C-fiber paucity in naked mole-rats. However, it should be noted that skin from β-*cat LOF* mice does not perfectly model the skin of naked mole-rats, most importantly because, whereas β-*cat LOF* mice lose hair follicles over time, naked mole-rat skin never contains hair follicles (with the exception of guard hairs and whiskers), so it may well be that the C-fibers that develop in the β-*cat LOF* mice are not dependent on hair follicles for their continuing survival. Nevertheless, it is striking that there is a small but significant loss of C-fibers but not A-fibers in the saphenous nerve of β-*cat LOF* mice. This observation is consistent with the idea that C-fibers that innervate hair follicles (Li et al.,^2011^; Wende et al.,^2012^) depend on the follicle for continued survival but that classical A-fiber mechanoreceptors do not.

In addition to certain A- and C-fibers being activated by noxious thermal stimuli, others are thermosensitive across a range that might be considered non-noxious (Iggo,^1969^). A thorough investigation of naked mole-rat A- and C-fiber thermosensitivity has not yet been described, but observations of huddling behavior and movement to warmer (heated) areas of cages suggests that naked mole-rats have thermoreceptors (our personal observations). Consequently, it seems unlikely that the C-fiber paucity in naked mole-rats reflects a loss of thermoreceptors, but only further investigation can fully answer this question.

### A-fiber characteristics in Bathyergidae

The three largest branches of the sciatic nerve are the tibial, common peroneal, and sural, and in the rat the number of A-fibers and C-fibers follows the order tibial > common peroneal > sural (Schmalbruch,^1986^). We found the same pattern across all six species, with the exception of the silvery mole-rat, in which there were more C-fibers in the sural nerve than in the common peroneal nerve (Tables 1, 2). It has also been documented in rat that lateral gastrocnemius nerves contain more A- and C-fibers than medial gastrocnemius nerves (Jenq and Coggeshall,1985a, b), and this was also observed here for all six species studied (Table 2).

In keeping with the presence of large-diameter motor neurons and type Ia sensory afferents in nerves innervating muscle (Sherrington,^1894^; Boyd and Davey,^1968^), we observed that A-fibers present in common peroneal, tibial, medial gastrocnemius, and lateral gastrocnemius nerves generally had larger diameters than A-fibers in saphenous and sural nerves, those fibers of the medial gastrocnemius nerve being the largest in every species (Fig. 3). The trend of muscle-innervating nerves containing larger-diameter A-fibers is not, however, fully apparent in the naked or Damaraland mole-rat: saphenous A-fibers do have smaller diameters than all other nerves, but sural nerves have diameters similar to those of both common peroneal and tibial nerves (Fig. 3, Tables 1, 2). In the rat sural nerve, ∼10% of myelinated fibers are motorneurons, innervating muscles in the foot (Peyronnard and Charron,^1982^), but it is possible that, in naked and Damaraland mole-rats, the percentage of sural A-fibers, which innervate muscle, is higher than in other species, giving rise to a larger average A-fiber diameter.

With respect to g-ratios, it has been calculated for myelinated nerves that the optimal ratio for conduction of current from one node to the next is 0.6 (Rushton,^1951^). Although higher average g-ratios have been observed in various A-fibers across different species (Williams and Chalupa,^1983^; Guy et al.,^1989^; Fraher and O'sullivan,^2000^), in the sciatic nerves in rodents g-ratios have been found to be closer to the theoretical optimum of 0.6 (Schwab et al.,^1984^; Sterne et al.,^1997^; Willem et al.,^2006^), but in other species they can be higher, for example, 0.8 in the European common frog *Rana temporaria* (Friede et al.,^1985^). In this study, we found that, as in other rodents, g-ratios in all branches of the sciatic nerve examined were ∼0.6 (Tables 1, 2). Size frequency distributions of A-fiber axon diameters in the saphenous nerve and other nerves showed a bimodal distribution in all mole-rat species (Fig. 4 for the saphenous nerve; other data not shown), which is likely reflective of Aα/β and Aδ fiber types, as has been previously observed in other rodents (Scadding,^1980^; Lynn,^1984^; Schwab et al.,^1984^; Schmalbruch,^1986^).

### C-fiber characteristics in Bathyergidae

Similarly to A-fibers, C-fiber diameter was generally positively correlated with species size (Fig. 5, Tables 1, 2). Furthermore, as with A-fibers, there was a trend for muscle innervating nerves to have larger C-fiber diameters (Fig. 5, Tables 1, 2). For the rat, Schmalbruch (1986) found there to be only a 0.03-μm difference in the means for C-fiber diameter in sural, common peroneal, and tibial nerves. In the present study, the range in the mean C-fiber axon diameter from sural, common peroneal, and tibial nerves was from 0.06 μm in the Damaraland mole-rat to 0.12 μm in the silvery mole-rat (Tables 1, 2). When it has been investigated in other rodent species, the C-fiber axon diameter distribution has been observed to be unimodal in saphenous (Scadding,^1980^; Alpsan and Lal,^1980^; Lynn,^1984^; Illanes et al.,^1990^), sural (Ochoa and Mair,^1969^; Schwab et al.,^1984^; Schmalbruch,^1986^; Hoffmeister et al.,^1991^), and common peroneal and tibial nerves (Schmalbruch,^1986^). We could confirm in every nerve, from all species examined, that there was a unimodal distribution for C-fiber diameter.

Although we observed a C-fiber deficit in naked mole-rat saphenous and sural nerves, we did not observe any difference in the number of C-fibers per Remak bundle (Fig. 7). This would suggest that factors known to be involved in normal Remak bundle formation, such as neuregulin-1 (NRG-1), function normally in naked mole-rats (Taveggia et al.,^2005^; Willem et al.,^2006^). Indeed, naked mole-rats apparently express high levels of NRG-1 in the nervous system throughout their normal life span (Edrey et al.,^2012^). Therefore, we can state that naked mole-rat C-fibers appear morphologically normal compared with those of the other species examined. It has long been known that NGF levels are lower in muscle tissues than they are in the skin (Korsching and Thoenen,^1983^; Shelton and Reichardt,^1984^; Lewin et al.,^1992^). It is thus possible that interference with NGF signaling in naked mole-rats might bring about a selective reduction of C-fiber in cutaneous nerves. However, the hypothesis that the C-fiber deficit observed in naked mole-rat cutaneous nerves is due to hypofunctional NGF-TrkA signaling should be tested more directly. One approach would be to clone and characterize the naked-mole rat NGF and TrkA genes to examine whether these proteins function differently compared with those of other rodent species. However, a detailed examination of the development of the sensory innervation of the skin in naked mole-rats, as has been conducted in the mouse (Crowley et al.,^1994^; Smeyne et al.,^1994^; Lechner et al.,^2009^), would be very difficult, given the eusocial nature of this species and the very long gestation time (∼75 days). It is still also possible that genes involved in the differentiation of sensory neuron lineages are also altered in the naked mole-rat in a way that leads to a selective C-fiber deficit in cutaneous nerves (Marmigère and Ernfors,^2007^).

## SUMMARY

We have shown that the naked mole-rat is unique within the family Bathyergidae in having a selective deficit in cutaneous C-fibers. We demonstrate that this deficit is unlikely to be fully accounted for by the naked mole-rat's lack of hair and hypothesize that hypofunctional neurotrophin signaling may be involved in producing the cutaneous deficit in C-fibers that we observed.
